# Spouse’s participation in perinatal care: a qualitative study

**DOI:** 10.1186/s12884-020-03111-7

**Published:** 2020-08-26

**Authors:** Nahid Mehran, Sepideh Hajian, Masoumeh Simbar, Hamid Alavi Majd

**Affiliations:** 1 School of Nursing and Midwifery, Shahid Beheshti University of Medical Sciences, Tehran, Iran; 2 Midwifery and Reproductive Health Research Center, School of Nursing and Midwifery, Shahid Beheshti University of Medical Sciences, Tehran, Iran; 3 Midwifery and Reproductive Health Research Center, School of Nursing and Midwifery, Shahid Beheshti University of Medical Sciences, Tehran, Iran; 4 Department of Biostatistic, School of Paramedical Sciences, Shahid Beheshti University of Medical Sciences, Tehran, Iran

**Keywords:** Spouses, Caregivers, Perinatal Care, Qualitative Research

## Abstract

**Background:**

Pregnancy is one of the most important periods of any woman’s life, wherein the support of her relatives, especially her spouse, enables her to tolerate the difficulties with good memories. However, in Iran, there are very few studies on the participation of spouses in the perinatal period. Therefore, the present study aimed to explain the concept of spouse participation in perinatal care.

**Methods:**

This is a qualitative study that was carried out in 2018 on spouse participation in perinatal care in Qom, Iran. Purposive sampling from pregnant or postpartum women, spouses, midwifery care providers, and key informants was performed according to study inclusion criteria. Semi-structured in-depth interviews were carried out until the data saturation was met. Also, the data analysis was performed based on a conventional content analysis approach according to Graneheim & Lundman steps using MAXQDA software (v.10). Five Guba and Lincoln criteria were applied to ensure the trustworthiness of data.

**Results:**

Fifty-three final codes were classified into 18 sub-categories, 7 categories, and 3 themes including empathy (emotional and cognitive understanding), accountability (supporting, position management, compassion), and consequences (help improvement of family function, improvement of maternal-neonatal health).

**Conclusions:**

Based on the findings of this study, the concept of men’s participation in this period has been defined as a set of empathic and responsive behaviors that can lead to improvement of the family function and mother and baby health.

## Background

Pregnancy is one of the most significant periods in every woman’s life that if is accompanied by the support of close relatives, especially their spouses, it will be easier to bear the difficulties [1]. The issue of spouses’ participation in women’s reproductive health care was addressed in the International Conference on Population and Development (ICPD) and the Fourth World Conference on Women [2, 3]. Despite the undeniable role of spouses in women’s reproductive health, it has historically attracted less attention [4].

Father involvement in pregnancy and childbirth has positive outcomes such as the reduced probability of preterm delivery, low birth weight, and fetal growth restriction [5–8]. Also, the men’s presence in maternal and child care opens up a new opportunity for health care providers to educate future fathers, and assist them in their health enhancement [9]. However, in some societies, the men’s awareness of their role in supporting their pregnant wives has been ignored and it results in direct outcomes (such as improper compatibility with the perinatal period and decreasing the father’s supportive role after birth [10–13]) and indirect negative impacts (such as educational, behavioral and developmental problems of children in terms of the reduction of father-child relationships, decrease in social support, and increased level of maternal stress hormones [14]). In many cases, although men are interested in engaging in pregnancy and childbirth, lack of incentives and restrictions and obstacles prevent their active participation [15]. Therefore, it seems necessary to strengthen the facilitator and remove the barriers as far as possible to increase the participation of men in perinatal care.

In Iran, in some health centers, spouses can attend in one to two sessions of eight-session classes for delivery preparation. However, there is a limited number of these training centers, and the proposed approach is not properly implemented and monitored [16]. In Qom city, Iran, there are limitations on the presence of men in midwifery units in most public centers. On the other hand, in Iran, especially in Qom, few studies have scrutinized the self-concept of spouse participation in maternal care. Since, the prioritization of mothers’ health and efforts to provide the desired services is a guarantee of the health of the family and the future generation and the provision of high-quality health services for pregnant women, as vulnerable groups, requires considering the roles and the experiences of women, men, and other practitioners, this qualitative study aims to explore the concept of spouse participation in perinatal care.

## Methods

### Study design

This qualitative research used a content analysis approach.

### Settings, sample, and recruitment

The participants included five women who were pregnant or had recent delivery, seven spouses, and nine key informants (deputy health managers and policymakers) of Qom city. Qom city is located 170 km to the south Tehran, Iran, with an area of 285 km^2^ and a population of 1,200,000 people. In Qom, due to religious conditions, there are different ethnicities from different parts of Iran and a few countries around the world.

The participants were selected through purposive sampling. The key informants were from public centers and the pregnant women and newly mothers and spouses were accessed through perinatal clinic or postpartum ward of Izadi hospital (one of the public hospital of Qom city). The inclusion criteria for pregnant or postpartum women (from one week to six months after delivery) and spouses consisted of the willingness to participate in this study, being Iranian, the ability to understand and express their experiences into Persian. Also, at least one year of working in midwifery- related units was added to the above criteria for caregivers and key informants. The exclusion criteria included the reluctance to take part in interviews and the withdrawal from participating in the study; however, no participant refused to be interviewed. The demographic characteristics of the participants are listed in Tables 1 and 2.

**Table 1:** The pregnant/ postpartum women and spouses’ demographic characteristics

Variable				
Age mean (years)		pregnant/ postpartum woman		34.7
		spouse		42.8
Age range (years)				29–60
Age group	pregnant/ postpartum woman		< 35 years	2
			≥ 35 years	3
	spouse		< 35 years	2
			≥ 35 years	5
Occupational status	pregnant/ postpartum woman		housewife	1
			employee	4
			Self-employment	0
	spouse		housewife	0
			employee	4
			Self-employment	3
Educational level	pregnant/ postpartum woman		Diploma or less	2
			Bachelor’s degree	2
			Master’s degree or higher	1
	spouse		Diploma or less	1
			Bachelor’s degree	5
			Master’s degree or higher	1
Number of children	pregnant/ postpartum woman		0	3
			1	2
			2	0
			3	0
	spouse		0	1
			1	1
			2	2
			3	3

**Table 2:** The key informants’ demographic characteristics

Variable		
Age mean (years)		38.3
Age range (years)		33–44 years
Age group	< 35 years	2
	≥ 35 years	7
Number of children	0	4
	1	2
	2	3
Educational level	Bachelor’s degree	5
	Master’s degree	3
	PhD	1
Field of Study	Midwifery	3
	Health Education	1
	Midwifery education	2
	Reproductive health	1
	Culture and communication	1
	History	1
Job	Midwife	2
	Faculty member	2
	cleric	2
	Head of Department of Maternal Health	1
	Expert of Family Health Department	1
	Teacher of birth preparation classes	1
Working place	Labor and Delivery Room (LDR)	2
	Maternal and Child Health Center (MCHC)	1
	Provincial Mothers Health Office	1
	Maternal Health Department of the Ministry of Health	1
	School of Nursing and midwifery	2
	Clerical Seminary	2
Work experience (years)	< 10	4
	10–20	2
	> 20	3

All of the interviews were conducted by the first author of this article (N.M) as a faculty member and Ph.D. student majoring in reproductive health with enough experience of qualitative research. Her main work’s experience has been taking care of pregnant mothers in public health centers in Qom. All of the steps for data recording and data analysis were taken under the supervision of the corresponding author (S.H) as a faculty member and Ph.D. in reproductive health with several years of qualitative research.

### Data collection

Data collection of this study was carried out between March and July 2018. At first, the needed permissions from the Deputy Chancellor of the Shahid Beheshti University of Medical Sciences and Deputy of Research of Qom University of Medical Sciences and voluntary verbal informed consent from participants were obtained, and the participants were selected according to the inclusion criteria. Prior to study commencement, the researcher explained the present study for participants to ensure their willingness to participate in the study and gave them face-to-face in-depth interviews either individually or pair wisely (depending on the desire of participants). Initially, one pilot interview was conducted, which was not analyzed, but it helped design the interview guide. The semi-structured questions of the interview were formulated by reviewing the literature and based on the experience of the author. Interviews began with an open question, such as “What is your perception of a spouse’s participation in prenatal, childbirth, and postnatal period? Please explain.” Then, as the interviews continued, more detailed questions were asked, such as “Do you have any experience in this regard? If yes, how was it?”, “In your opinion, how much can a spouse’s participation be effective during pregnancy, childbirth process, or postpartum period?” etc. (see Table 3). The interviews were recorded using a tape recorder and then transcribed at the right time to document the data. During the interviews, observations and field note method was implemented and non-verbal data such as tone and gestures were recorded. The interviews lasted for 30–90 min (average of 55 min) in the Izadi hospital (one of Qom’s public hospitals, located almost in the center of the city with many clients) and participants’ homes — or the places where the participants felt more comfortable.

**Table 3:** Interview guide during the face-to-face interviews with participants

**Trigger question of pregnant/postpartum women and spouses and key informants**: 1. What is the meaning of spouse’s participation in prenatal period, in your mind? 2. What is the meaning of spouse’s participation in childbirth period (since beginning the labor pain until discharge of hospital after birth), in your mind? 3. What is the meaning of spouse’s participation in postnatal period, in your mind?
Continue questions: **A. Continue questions related to pregnant/postpartum women**: 1. Do you have any experience with your spouse in your past or current pregnancy or delivery? 4. In your opinion, how was this experience? 5. In your opinion, how much can your spouse’s participation be effective during pregnancy, childbirth or after it? 6. In your opinion, what does your spouse need to increase his participation with you, during this period? 7. In your opinion, what are the current obstacles for your spouse’s participation during perinatal period? 8. In your opinion, which factors can increase your spouse’s participation during perinatal period? **B- Continue questions related to spouses**: 1. Do you have any experience of participation with your wife in past or current pregnancy or delivery? 2. In your opinion, how was this experience? 3. In your opinion, how much can your participation be effective during pregnancy, birth process or postpartum? 4. In your opinion, what do you need to increase your participation during this period? 5. In your opinion, what are the current obstacles for husband’s participation during this period? 6. In your opinion, which factors increase husband’s participation in this period? **C. Continue questions related to key informants**: 1. Do you have any experience with spouse’ participation in perinatal period? 2. In your opinion, how was this experience? 3. According to your job experiences, how much can spouse’s participation be effective during perinatal period? 4. According to your job experiences, what do husbands need to increase their participation with their wife during this period? 5. According to your job experiences, what are the current obstacles for spouses’ participation during this period? 6. According to your job experiences, which factors increase spouses’ participation in this period?

Key informants of this study were the deputy health managers at the Qom University of Medical Sciences and policymakers from the Ministry of Health and Medical Education. The data collection of key informants was similar to the former process used for spouses. In this respect, they were semi- structurally interviewed deeply with the same open questions for 45–90 min (average of 60 min) in hospitals, health centers, or other places. The questions guide of interviews is shown in Table 3.

The interviews with the participants continued until the occurrence of data saturation, i.e., new data entering the study did not alter the available classification and not suggest the creation of a new class [17]. Data saturation of this study was obtained in the 15th interview. Nevertheless, six more interviews were conducted to ensure the reliability of data collection. Besides, no interviews needed to be repeated.

### Data analysis

**Fig. 1:**
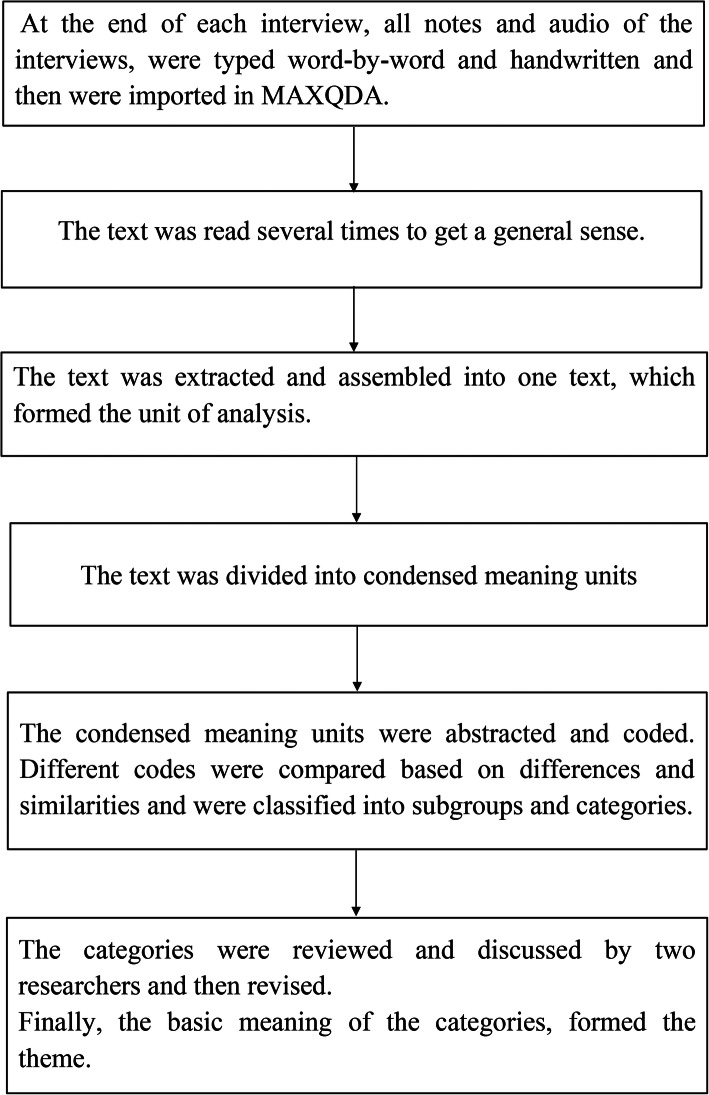
Flow chart of Graneheim & Lundman analysis (2004)

The data were analyzed using the conventional content analysis method, according to Graneheim & Lundman [18]. Accordingly, at the end of each Persian-based interview, all notes and the audio file of the interviews were word-by-word typed and handwritten. Then the typed texts were read several times to get an overview of their contents. Based on the inductive method, semantic units and initial codes were determined, the similar codes were embedded in more sub-categories, and the categories and themes appeared [18]. For better data management, after recording on the paper, MAXQDA v.10 was used simultaneously with each interview. MAXQDA is a software program designed for qualitative and mixed methods data, multimedia and text analysis. The program’s central elements are the systematic assignment (“coding”) of data segments (text, tables, media …) to major themes (“codes”) and the possibility of taking notes of references, ideas, etc. directly in the text (“memos”) (https://www.maxqda.com/how-to-analyse-qualitative-data). The analysis stages are shown in Fig. 1.

### Rigor and trustworthiness

The five Guba and Lincoln criteria (including credibility, dependability, transferability, conformability, and authenticity) were applied to ensure the trustworthiness of data [19]. To increase the credibility of the data, “searching for disconfirming evidence” was done to extract the data that challenged the conceptualization and descriptive theory extracted from the data. Therefore, it was attempted to make a comprehensive sampling of people who could have opposing views via selecting a variety of participants with different characteristics (age, educational level, occupation, number of pregnancies, live children, and so forth). Also, the codes were independently reviewed by other members of the research team. Besides, the prolonged engagement of the researcher was noticed as well.

The interviews were carefully recorded and written to verify the dependability of the data. Also, during writing the report, it was cited to participants’ conversations. Also, the study was reviewed by the supervisors and experts, and some interviews were randomly re-coded over the next two weeks to ensure coding consistency.

A rich and detailed description of the research process was provided to increase the transferability so that readers of the report can understand the steps and interactions of the study.

The opinions of three experts in qualitative research and reproductive health were also regarded to increase the conformability of the data.

The researchers made efforts to select the appropriate people for interviews and provide a rich and detailed description to increase the authenticity of the data [20].

### Ethical considerations

The Ethical approval of this research was received from the Ethics Committee of Shahid Beheshti University of Medical Sciences (ethical approval code: IR. SBMU.PHNM.1394.284). In this regard, the following considerations were incorporated in the present study: obtaining the voluntary verbal informed consent, preservation of anonymity, paying attention to the willingness of participants to choose the location and time of the interview, charging no fee to the participants, preservation of confidentiality, and recognizing the right of participants to leave the study at any time.

## Results

At the end of the interview and data saturation, 1856 initial codes were extracted. Because many of the resulting codes were similar, the same codes were merged. At last, 53 final codes were classified in 18 sub-categories, 7 categories, and 3 themes. The final themes were empathy, accountability, and consequences (Table 4).

**Table 4:** Results of data analysis

Code	Sub-category	Category	Theme
Understand the situation	Empathetic attention	Emotional understanding	empathy
Pay attention to the needs of the spouse and child			
Control of feelings and emotions			
Follow up care			
Staying in empathic situations			
Receive the feelings of the wife	Encouraging and making hope		
Displaying enthusiasm and proper feedback to the spouse			
Induction of faith			
Reassuring			
Compatibility with spouse position	sacrifice		
Reduce expectations and demands			
Resiliency Exercise			
Pre-pregnancy preparation	Readiness	Cognitive understanding	
Pregnancy Preparation			
Preparations during and after childbirth			
Commitment	Responsibility		
Lack of deposit of responsibility to others			
Trying to eliminate negative beliefs	Reforming Attitudes		
Creating positive beliefs			
Participation in household chores	Tangible support	supporting	accountability
Participation in the care of children			
Material support			
Trying to get information related to the situation	Information support		
Applying the correct information in related situations			
Planning before pregnancy	Planning	Position management	
Planning for birthday			
Managing the exposure to unexpected situations	Management		
Creating balance between indoors and outdoors			
Tension management			
Positive interventions in risky situations	Proper interaction	Compassion	
Action to reduce the spouse’s suffering and worries			
Active participation in prenatal care	Dynamic presence		
Accompany during childbirth			
Active participation in postpartum care			
Intellectual intimacy	Intimacy	Help improve family function	consequences
Sexual intimacy			
Emotional intimacy			
Timely intimacy			
Spiritual intimacy			
Psycho-emotional security	Security		
Economic security			
Communication security			
Flexibility	Solidarity		
Correlation			
Maintaining the dignity in the family	Respect		
Maintaining the social position			
Self-esteem			
Mental health	Maternal health	Improve maternal-neonatal health	
Physical health			
Social health			
Secure attachment	Neonatal health		
Desirable evolution			
Desirable growth			

### Empathy

Men should understand their wives, either emotionally or cognitively. This theme contained 2 categories and 6 sub-categories.

#### Emotional understanding

Most participants believed that spouses should pay attention to their wives, encourage them to be hopeful about the future, and, if necessary, make sacrifices. This category had three sub-categories.

#### Empathetic attention

Some participants stated that a spouse should understand the new situation of his wife during pregnancy, childbirth, and the postpartum period. Also, he should be mindful of the needs and desires of his wife and children to be able to fulfill them.

*“I can understand that in the situation that she is in, she may sometimes be psychologically disruptive, speak up, and get offended. I should understand her position.” (Participant No.7, spouse, group* *≥* *35 years).*

*“Not to tell me, the others or my wife want to tell me to do it. I myself must understand what is better to do.” (Participant NO.10, key informant, group* *≥* *35 years).*

Some participants believed that a spouse should be able to control his feelings and emotions in this period and should not transfer them to his wife. He should be mindful of midwifery care of his wife during pregnancy, childbirth, and the postpartum period and follow it up with more attention. He should occasionally attend cares, meetings, classes, and maternity programs to better understand his wife.

#### Encouraging and giving hope

Some participants believed that a spouse should show his empathetic attention to the wife by asking for her condition, whether through in-person speaking or by telephone or from a person accompanying his wife.

*“When she is admitted to hospital, he should be in regular contact with her” (Participant NO.13, Female, key informant, group* *≥* *35 years).*

Spouses should be able to respond appropriately to the wife’s feelings and behaviors during this period, encourage her, and give her hope by showing his enthusiasm for the birth of the child. Some participants referred to the role of a spouse in restoring the wife’s faith through spiritual conversations.

*“Whenever I said to him: “I’m worried,” He said: “Trust in God,” and this phrase pleased me” (Participant NO.12, Pregnant woman, group* *≥* *35 years).*

#### Sacrifice

Some participants believed that a spouse should adapt himself to the wife’s conditions until this period ends well as a good memory in her mind. Also, he should not have the same previous expectations in pregnancy and, even more importantly, in the postpartum period. He should lower expectations and practice to raise his tolerance towards the temporal changes of his wife’s behaviors in this period.

*“I had a bad nausea, so I even hated smells, even my husband’s smell. He was very cooperative with me.” (Participant No.13. female, key informant, group* *≥* *35 years).*

### Cognitive understanding

From the viewpoint of most participants, spouses should logically understand their wives, be accountable, and have a positive attitude towards pregnancy and postpartum periods. This category contained three sub-categories of readiness, accountability, and reforming attitudes.

#### Readiness

Some participants believed that when a wife and her spouse decide to have a baby, the spouse needs to get ready to start the fatherhood process. He should increase his information, get familiar with the signs of risky situations, and handle the required affairs. Moreover, at the end of pregnancy, he should be prepared for the birthing of a new family member.

Some key informants stated that the responsibilities of spouses do not merely culminate with the childbirth process and hospitalization of their wives, but rather they should show their empathy by the presence at the hospital and fulfillment of the needed actions for delivery and discharge of his wife and child from the hospital.

*“If men can attend prenatal classes, they will have some awareness of what they should do when they are at home, when their wife has problems” (Participant N0.1, Spouse, group* *≥* *35 years).*

*“It is necessary to provide the necessary conditions for the return of his wife to home. Perhaps one of the things that worries the ladies to go back to the home is that they face a cluttered house when they return home” (Participant NO.5, Female, key informant, group* *≥* *35 years).*

#### Responsibility

Some participants said that a spouse should be familiar with his duties and responsibilities during this period. He should not expect others to take on his responsibilities.

*“This is not rational that I put everything on my wife’s shoulder” (participation NO.3, Spouse, group* *≥* *35 years).*

*“He should not take his responsibilities off on other women’s shoulders, although it’s possible that they are tired because of making meals or hosting the guests.” (Participant NO.12, male, key informant, group* *≥* *35 years).*

#### Reforming Attitudes

Some participants acknowledged having positive attitudes towards pregnancy, childbirth, and postpartum as one of the responsibilities of spouses during this period and believed that they should think of pregnancy and childbirth as a mutual role. If they have a positive attitude and be familiar with the problems of this period, they will not find their wives’ behavioral changes spoiling. Also, they should try to correct the negative and false believes of the people around them.

*“Even about the false words that people around tell her, for example, recommending a herbal medicine for curing newborn jaundice or colic, he should not let his wife take that wrong herbal medicine” (Participant NO.4, female, key informant, group* *≥* *35 years).*

### Accountability

Most participants believed that, during this period, in addition to emotional and cognitive understanding, spouses should support their wives and show their compassion through proper management and correct planning. In this regard, they should also take the needed actions to reduce their wives’ discomfort and handle dangerous situations. This theme contained 3 categories and 6 sub-categories.

#### Supporting

Some participants believed that a spouse should support his wife in home care, taking care of newborn children and other children, and financial affairs. He should also increase information and awareness and acquire the ability to use the information properly. This category contains two sub-categories, namely tangible support and information support.

##### Tangible support

All participants believed that spouses should be involved in housework, especially those activities difficult to do for a pregnant woman. They said that the participation of spouses in the postpartum period is more important than ever before due to the addition of newborn care to her previous activities.

*“After childbirth, we should help in all aspects, especially in the first 30–40 days, which is very difficult.” (Participant NO.1, Spouse, group* *≥* *35 years).*

Some participants acknowledged the financial support of wife and children and meeting the living expenses and material needs as spouse’s duties.

##### Information support

Some participants emphasized the necessity of men’s awareness of pregnancy, childbirth, and the postpartum period as a spouse’s duties and complained of insufficient awareness in this area. Also, men should be able to use this information in appropriate situations. In other words, this higher and desirable level of participation develops after cognitive understanding, i.e., at the cognitive understanding stage, the spouses’ knowledge was increased and in this stage, they utilized that learned information.

*“When wife’s labor pains start, her spouse can remind her, the breathing techniques since she is not focused.”(Participant NO.13, female, key informant, group* *≥* *35 years).*

#### Position management

Proper planning for childhood and well-suited management to deal with the situations and challenges of this period were other statements made by the participants. This category contained 2 sub-categories: planning and management.

##### Planning

Some participants believed that spouses should plan before childbearing, i.e., when they decide to have children. They identified the need to plan for a new family member’s birthday as one of the spouse’s responsibilities during this.

##### Management

Some female participants said that a spouse should be able to manage unexpected situations, such as the sudden onset of labor pain or the occurrence of risk signs, and he should not cause the wife’s discomfort. Almost all female participants believed that spouses should make a balance between indoor and outdoor activities and not prefer their jobs to the family.

*“This psychologically and culturally is needed to receive the attention that “we work to live, not we live to work.” Men must understand that the value of life is something rather than money.” (Participant NO.17, Pregnant woman, group < 35 years).*

#### Compassion

Compassion is indeed an understanding of the problems, as well as having the duty to help solve the problems of ourselves or others, including key components such as altruism, kindness, and joy. In general meaning, compassion might be confused with empathy, whereas empathy is a stage before compassion (prerequisite) [21]. This category contains two sub-categories: proper interaction and dynamic presence.

##### Proper interaction

Some key informants believed that spouses should be able to take necessary actions in dangerous situations, including dialogue and interaction with health care providers and giving them the needed guidance to manage the situation accurately.

*“If she is at high risk and needs special care, f (e.g., special dietary care or certain medication orders), her spouse can interact with her midwifery/doctor” (Participant NO.12, male, key informant, group* *≥* *35 years).*

##### Dynamic presence

Some participants stated that spouses should actively participate in prenatal care. After sending her to the care centers, the spouse should refer to the doctor/midwife, if possible, and listen to their recommendations and discuss the conditions of his wife with him/her to better meet her needs. Also, in this case, he can enjoy hearing the fetal heart.

*“When I was going to receive care, he came inside, wherever allowed, and talked with my doctor.” (Participant NO.14, Female, key informant, group < 35 years).*

Although there is no possibility of active participation of spouses in childbirth process in many birth centers of our country, especially in state centers, most of the participants regretted this issue and stated that the spouse should be with his wife and give her encouragement and comfort when he is present in the labor room/operating room. If it is not possible, the spouse should be in contact with the person accompanying his wife or with her assigned midwife and informed of his wife’s status. Some participants suggested that the center’s conditions should provide the possibility that the spouse is the first person meeting his wife after childbirth. Also, some participants believed that spouses should also accompany their wives and newborns in postnatal care and play an active role.

*“After childbirth, he should carry his baby for neonatal cares, such as thyroid screening.”(Participant NO.5, Female, key informant, group* *≥* *35 years).*

### Consequences

From the participants’ standpoint, men’s participation in perinatal care has positive outcomes, such as helping the improvement of family function and maternal/neonatal health. This theme contained two categories and six sub-categories.

#### Help with the improvement of family function

The participants referred to creating and enhancing intimacy, sense of security, coherence, and respect among family members as positive outcomes of spouse’s participation in this period. This category had four sub-categories.

##### Intimacy

Intimacy is the ability to develop deep relationships among couples to resolve conflicts, share the experiences, and receive a sense of internal security from the other side.

Some participants believed that spouse participation leads family members, especially couples, to interact with each other more likely through exchanging views and information. Furthermore, they will be similar in attitudes and respect the opinions of one another (intellectual intimacy). Also, the husband and wife become sexually closer (sexual intimacy), productive talks between couples increase, and they feel well emotionally, resulting in their physical and mental health (emotional Intimacy). Furthermore, the spouse spends more time with his wife and children (time intimacy), and a sincere spiritual relationship might be built between the couple as well (spiritual intimacy).

*“When a woman shares her plan with her spouse, she gets more energy, can pay attention to her body, and is effective in the marital and sexual relationship. She is not tired.” (Participant NO.16, female, key informant, group < 35 years).*

##### Security

Some female participants attributed the feelings of reliance on the spouse, peace of mind of wife and children, and financial comfort to spouse’s efforts to meet the financial needs of his family and better communication of family members, particularly the relationship between the father and his children as a consequence of spouse participation.

*“If a spouse has empathy and cooperation, then the woman is warmly backed.” (Participant NO.1, Spouse, group* *≥* *35 years).*

##### Solidarity

Solidarity is the feeling of correlation, bondage, and emotional commitment that members of a family have toward one another [22]. Some participants assumed the spouse’s participation as a factor for causing greater solidarity among family members. They believed that participating spouses have more flexible behaviors.

*“For my second baby, I was going to bring her to the Valiasr Hospital for birthing, but she said that she wants to go to the Izadi Hospital. Although I did not agree, I brought her to the Izadi hospital.”(Participant NO.7, Spouse, group* *≥* *35 years).*

##### Respect

Some male participants stated that if a spouse is involved in his wife’s pregnancy, childbirth, and the postpartum period, she feels that she has a good situation and dignity in life. Also, it would preserve and enhance the social status of children in the future. Some participants believed that the participation of men helps maintain and increase the self-confidence of his wife and children.

*“If you sometimes pull the back of your wife’s neck up with your hands, your children’s self-esteem will rise. In general, the child’s personality mainly forms in the house.” (Participant NO.1, Spouse, group* *≥* *35 years).*

#### Improvement of maternal-neonatal health

In addition to improving the function of the family, the participation of spouses helps promote maternal and infant health.

##### Maternal health

Some participants stated that the participation of spouses improves physical and mental health. Furthermore, peace of mind, the feeling of not being alone, and having secure and reliable support in the face of social problems improve the wife’s social health.

*“If a spouse participates at home, his wife will rest further, become healthy in a shorter time, and her stitches will get better sooner.” (Participant NO.19, Spouse, group* *≥* *35 years).*

*“When a woman sees her spouse at her side, doing everything to ensure her comfort, she will surely feel peace and convenience. She feels that she is backed, and there is someone that can help her in difficult circumstances, and she is not alone.” (Participant NO.5, female, key informant, group* *≥* *35 years).*

##### Neonatal health

Some participants stated that spouse participation develops a deeper emotional relationship between mother and baby and makes a secure attachment. Also, it might have a positive effect on the child’s developmental process, especially on psychological and emotional development.

*“If the father is involved in the caring process of his or her child ... it will certainly affect the psychological development of the children”. (Participant NO.5, female, key informant, group* *≥* *35 years).*

## Discussion

The present qualitative study was conducted to determine the concept of spouse’s participation in perinatal care. According to the obtained results, the most important aspects of male participation in perinatal care were empathy, accountability, and consequences. As a general result, the concept of spouse’s participation in prenatal care, childbirth, and postpartum period has been defined in a set of empathic and accountable behaviors towards their wives based on emotional and cognitive responses, position management, support, and compassion, that can lead to favorable consequences such as the improvement of the family function and mother and baby health.

The participants believed that a spouse should empathize with her wife and understands her during the perinatal period, emotionally and cognitively. In several studies, the necessity of loving and empathetic attention has been regarded as the most important aspect of a spouse’s participation in perinatal care [2, 23, 24]. The empathy — described as the ability to supportively communicate a sensitive awareness and respect another person’s feelings— helps the development of mutual trust shared understandings and, in turn, the development of a fundamental quality in any helping relationship [25]. Men and women have realized that spouses are the best providers of their wives’ emotional needs in the perinatal period [26]. Ergo’s study demonstrated that a spouse’s emotional support is the most influential factor in decreasing postpartum depression [27].

Furthermore, participants mentioned accountability as another aspect of a spouse’s participation, which is defined as being accountable to a person for the expected performance. It differs from the responsibility that is an intrinsic obligation and commitment of the individual to perform all the activities assigned to him/her [28]. The participants believed that a spouse should be accountable to his wife for his behaviors during this period. He should have proper interaction and dynamic presence, support her wife, plan and manage the hazardous and non-hazardous situations. Also, participants in a study conducted by Firouzan et al. emphasized the comprehensive participation of spouses in married life (e.g., housework, cooking, and care of children) and all decision-making during the perinatal period, which are in line with the present study. This outcome could be due to the increase in females’ awareness of their rights in married life and their employment and contribution to the household economy [29]. Moreover, the participants believed that spouses should be prepared for safe delivery and unexpected events in the perinatal period. He should consult with his wife about the place of delivery, transport her to the hospital on time and accompany her, and stay in the delivery room or, if not allowed, in the hospital until birthing of the child. Most participants preferred spouses’ physical presence in the delivery room according to the results of Kaye et al. and Simbar et al. [24, 30]. However, despite the distinct Islamic recommendations emphasizing the spouse task in supporting his wife, men are not allowed to be present in the delivery room due to some cultural beliefs, negative attitudes of staffs, inadequate personnel, heavy workload, and management structures of most hospitals, especially in public centers. This issue has highlighted the lack of spouses’ participation in the delivery period in the participants’ minds of this study. Fortunately, according to the Ministry of Health and Medical Education recommendations for the presence of spouses during labor in mother-friendly hospitals in recent years, most hospitals are moving towards modifying the delivery units’ structures to meet this goal. However, these facilities are available in all health centers.

Helping improve the family function and maternal-neonatal health caused by the participation of spouses is another area that was stated by most women and men of this study, which is consistent with the results of the study carried out by Simbar et al. and Davis et al. [24, 31]. Although the benefits of men’s participation in perinatal period have been acknowledged in various studies [29, 31–35], it should be noted that pregnancy can be the most stressful period for men undergoing the transition to parenthood, especially in terms of own psychological reorganization [36, 37]. Their involvement in partners’ pregnancy may indicate that they would like to have an important role in prospective child’s development [38], but in this way, they may experience various levels of mood changes and anxiety due to fear of insensibility, past events, the transitional changes to parenting and work-related problems [39] and this can lead to mental health problems for them if not diagnosed early [40].

As a general result of this study, the concept of spouse’s participation in prenatal care, childbirth, and postpartum period has been defined as a set of empathic and accountable behaviors towards their wives based on emotional and cognitive responses, position management, support, and compassion, that can lead to favorable consequences such as improvement of the family function and mother and baby health.

Since the presence of spouses in the midwifery care unit plays an important role in increasing their participation in the perinatal period, it is recommended that health care providers allow spouses, if wives wish, to attend and participate in their perinatal cares. However, this needs to change the attitude of health managers and staff towards the presence of men and improve the physical structure of health centers.

Furthermore, since some spouses do not have the proper knowledge about participation in the perinatal period, despite the desire to do that, it is suggested that health centers provide training classes in this regard, with the minimum cost, especially on holidays. Therefore, spouses taking part in these classes could participate in the prenatal period more effectively, contribute to the improvement of the health of their wives and child, and promote the health of the whole family as a result.

To our knowledge, this was the first study in Qom city that attempted to explore spouse’s participation during perinatal period. There was a need for a conceptualization of male involvement in this period because it is an important concerns of women who suffer from little husbands’ support. The findings are useful to them and to health policy makers and managers to programming for resolving this issue.

Another strength of this study is almost comprehensive study that the data generated from multiple respondents including pregnant or recently-delivered women, spouses and health providers and managers.

Despite the diversity of participants in this study, considering its qualitative approach, the obtained results can not be generalized to other places and cultures. However, the results may be beneficial to those willing to use the results while considering the limitations.

## Conclusions

The present study was conducted to determine the concept of spouse’s participation in perinatal care. Based on the findings of this study, the concept of men’s participation in this period has been defined as a set of empathic and responsive behaviors towards their wives based on emotional and cognitive responsiveness, position management, support, and compassion that can lead to desirable consequences such as the improvement of the family function and mother and baby health.

## Data Availability

The datasets used and/or analyzed during the current study are available from the corresponding author on reasonable request.

## Abbreviation

ICPD: International Conference on Population and Development.
